# Cancer Incidence and Mortality in Firefighters

**DOI:** 10.31557/APJCP.2020.21.3.575

**Published:** 2020-03

**Authors:** Swaantje Casjens, Thomas Brüning, Dirk Taeger

**Affiliations:** *Institute for Prevention and Occupational Medicine of the German Social Accident Insurance, Institute of the Ruhr University Bochum (IPA), Bochum, Germany. *

**Dear Editor**

We have read with interest the review and meta-analysis of cancer incidence and mortality of firefighters in this journal (Soteriades et al., 2019). Nevertheless, we are wondering why the literature search ends more than a decade ago. Since January 2007 about 30 studies and three meta-analyses (Jalilian et al., 2019; Crawford et al., 2017; Sritharan et al., 2017) on the association between firefighting occupation and cancer risk have been published. For example, the omitting of the latest studies might have lead to an underestimation of the risk of skin melanoma compared to the latest meta-analysis (Jalilian et al., 2019). Possibly, additional preventive medical checkups and screenings for firefighters might have led to a higher rate of diagnosed malignant melanoma of the skin in the recent past.

Furthermore, we do have some remarks regarding the methodological approach of the presented analysis. As stated before, cohort studies differ from population based case-control studies (Casjens et al., 2019). The latter are not always found by a formalized search strategy because not all studied occupations of a paper are indexed. Hence, the pooling of different risk estimates such as SMRs, SIRs and ORs is not appropriate and might lead to biased results. We suggest to analyze cohort and case-control studies separately. 

Examing the difference in results based on study quality is an interesting approach. However, we are missing the information on the assigned quality to each study. This could be easily added to the tables in appendix A which unfortunately is incomplete (eight included studies are not listed). Several scales to assess the study quality exist and produce apparently divergent results as almost every study assessed by the Newcastle-Ottawa Scale has “good” quality (Jalilian et al., 2019). This rises the question if study quality can be measured by simple scales thoroughly (Stang, 2010).

Lastly, the authors did not clearly state which type of random effects model was used. Presumably, the commonly used DerSimonian-Laird estimators were calculated although simulation studies suggest that better alternatives exist to estimate the between-study variance (Veroniki et al., 2016).

Anyway, we agree with Soterides et al., (2019) that the exposures for firefighters have changed over the past decades and that we need more studies with better assessments of today’s exposure. Due to the special nature of this occupation these studies do not exist so far.

## Conflict of interest declaration

The authors declare no conflicts of interest.
